# Association between osteoporosis and gallstone based on the National Health and Nutrition Examination Survey (NHANES) 2017–2020: a cross-sectional study

**DOI:** 10.3389/fpubh.2025.1562984

**Published:** 2025-04-08

**Authors:** Chuang Li, Qifan He

**Affiliations:** ^1^Department of Interventional Radiology, The First Affiliated Hospital of Soochow University, Suzhou, China; ^2^Department of Radiology, Haining People’s Hospital, Jiaxing, China

**Keywords:** osteoporosis, gallstone, older adults, NHANES, survey

## Abstract

**Background:**

Osteoporosis and gallstones are both common conditions in older adults, yet the association between them remains unclear. This study investigates the relationship between osteoporosis and gallstones in a large, nationally representative sample of U.S. adults using data from the National Health and Nutrition Examination Survey (NHANES) 2017–2020.

**Methods:**

7,766 participants aged 50 years or older with complete osteoporosis questionnaire or bone mineral density (BMD) data included in the study. Osteoporosis status was determined based on self-reported physician diagnosis and femoral neck BMD measurements. Logistic regression models were used to examine the association between osteoporosis and gallstone risk, adjusting for sociodemographic, lifestyle, and health-related covariates. Generalized additive models (GAM) and smoothing curve fitting were used to explore the non-linear relationship between femoral neck T-scores and gallstone prevalence. Mediation analysis was performed to investigate the roles of serum calcium and phosphorus in mediating the osteoporosis-gallstone association.

**Results:**

Our analysis revealed a significant association between osteoporosis and an increased risk of gallstones, particularly among individuals aged 65 and older, non-Hispanic whites, those with a college education or higher, and those with comorbid conditions such as hypertension and diabetes. In multivariate logistic regression models, individuals with osteoporosis had a higher risk of gallstones compared to those without osteoporosis (OR: 1.52, 95% CI: 1.15–1.99, *p* < 0.001). Further analysis based on femoral neck BMD indicated that osteoporosis (T-score ≤ −2.5) was significantly associated with an increased risk of gallstones (OR: 1.67, 95% CI: 1.11–2.46, *p* = 0.01). Generalized additive model analyses revealed a nonlinear relationship between femoral neck T-scores and gallstone prevalence. Mediation analysis indicated that serum calcium and phosphorus partially mediated the association between osteoporosis and gallstones.

**Conclusion:**

This study demonstrates a significant association between osteoporosis and an increased risk of gallstones in older adults. Our findings highlight the importance of routine gallstone screening for individuals with osteoporosis, particularly those with additional risk factors. Femoral neck BMD may serve as a more effective marker of gallstone risk than lumbar spine BMD.

## 1 Introduction

Osteoporosis is a chronic, systemic skeletal disorder characterized by reduced bone mass and deterioration of bone tissue microarchitecture, resulting in an elevated risk of fractures (1). The treatment and prevention of osteoporosis have become significant public health concerns worldwide (2). The global prevalence of osteoporosis is estimated at approximately 18.3% (3, 4), with around 14.1 million adults aged 50 years and older in the United States affected (5, 6). Osteoporosis may also be linked to metabolic disorders beyond bone health, including gallstones, due to shared risk factors such as aging, hormonal changes, and chronic inflammation.

Gallstones are a prevalent digestive disorder in the United States, affecting approximately 10–15% of adults and contributing over $600 million annually to healthcare costs (7). Most individuals with gallstones remain asymptomatic; however, without timely screening, gallstones can progress to symptomatic forms, leading to complications such as acute or chronic cholecystitis, pancreatitis, or even gallbladder cancer (8). This progression places a substantial strain on both individual health and public healthcare resources.

Although osteoporosis and gallstones are both prevalent in older populations, research investigating their association is limited. Only one Taiwan study has linked heavy metal exposure to gallstones but it lacks generalizability and omit key variables such as bone density, blood calcium, blood phosphorus, and BMI, potentially affecting the reliability of findings (9). It remains unclear whether individuals with osteoporosis have a heightened risk of developing gallstones compared to those without osteoporosis. This study utilizes large-scale, nationally representative data from the United States to investigate the association between osteoporosis and gallstones.

## 2 Methods

### 2.1 Study population

This study utilized data from the National Health and Nutrition Examination Survey (NHANES), a comprehensive, biennial health assessment managed by the National Center for Health Statistics (NCHS). NHANES integrates interviews, physical examinations, and laboratory assessments to evaluate the health and nutritional status of the U.S. population, encompassing demographic and health data across age, gender, and race/ethnicity. For this study, data were drawn from the NHANES cycles spanning 2017–2020, which predate the COVID-19 pandemic and include data collected through March 2020.

**Figure 1:**
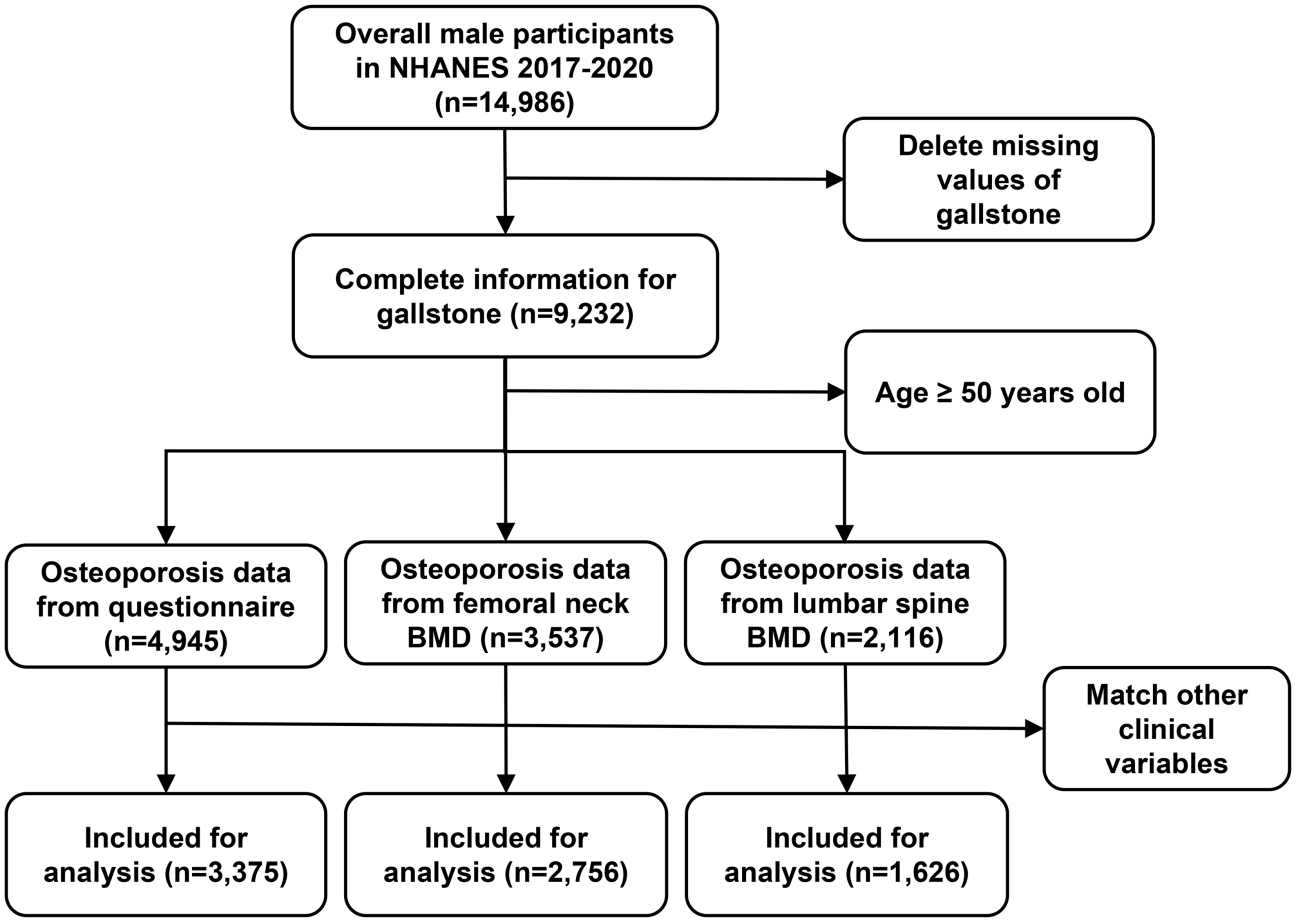
Flowchart of data sets preparation.

Inclusion criteria for this analysis were as follows: (1) age ≥ 50 years; (2) questionnaire data confirming gallstone presence; (3) complete data on osteoporosis status or bone mineral density (BMD); and (4) available data on smoking history, vigorous recreational activity, and additional covariates (Figure 1).

Individuals with missing data on key variables, such as osteoporosis status, gallstone history, and essential covariates (e.g., BMI, smoking, and diabetes), were excluded from the analysis. Additionally, participants with severe chronic liver disease or other metabolic bone disorders were excluded to minimize potential confounding.

### 2.2 Osteoporosis history and BMD data

Osteoporosis status was ascertained using NHANES questionnaire responses, wherein participants were asked, “Has a doctor ever told you that you had osteoporosis, sometimes referred to as thin or brittle bones?” Affirmative responses indicated a history of osteoporosis. The survey on osteoporosis was conducted by trained interviewers at home, and a computer-assisted personal interview (CAPI) system was utilized. We acknowledge the reliance on self-reported physician-diagnosed osteoporosis and gallstones as a study limitation, as it may introduce recall bias. To address this, we conducted sensitivity analyses using BMD-based osteoporosis classification as an alternative measure.

Bone mineral density (BMD) measurements were conducted using dual-energy X-ray absorptiometry (DXA), which is considered the gold standard for assessing bone density (10). The DXA examination protocol is comprehensively outlined in the Body Composition Procedures Manual available on the NHANES website. The T-score is a standard measure used in bone density testing to assess bone health and determine osteoporosis risk (11). T-scores were calculated as follows: T-score = (BMD - reference BMD) /reference SD. In line with World Health Organization (WHO) guidelines (12), we utilized non-Hispanic white females aged 20–29 years from NHANES III data as the reference group for femoral neck measurements (13), while lumbar spine references were derived from the Vital and Health Statistics published by the Centers for Disease Control and Prevention (CDC) (14). A T-score of ≤ −2.5 indicated osteoporosis, whereas a T-score between −2.5 and − 1 was classified as osteopenia. In addition, all participants were grouped according to quartile of T-score (Q1: 75–100%; Q2: 50–75%; Q3: 25–50%; Q4: 0–25%), with the highest T-score group (Q1) as the reference.

### 2.3 Definition of gallstones

Gallstone presence was determined by participants’ responses to the question, “Has a doctor ever informed you that you have gallstones?” Affirmative answers indicated gallstone presence, while negative answers indicated the absence of gallstones.

### 2.4 Covariates

Covariates were selected based on previous studies and NHANES data availability. Confounding variables included demographic, examination, laboratory, and questionnaire data covering age, gender, race, educational level, body mass index (BMI), serum calcium levels, physical activity, drinking history, smoking history, diabetes, and hypertension. Race was categorized based on NHANES classification standards. Education level was grouped into three categories. Total calcium levels were extracted from NHANES laboratory data. Physical activity status was assessed by responses to questions about engaging in continuous, vigorous-intensity activity for at least 10 min. For drinking status, participants were asked, “How often have you consumed alcoholic beverages in the past 12 months?” Those reporting consumption of less than once per month were categorized as non-drinkers. Diabetes and hypertension were classified according to self-reports of prior diagnoses by a medical professional.

The selection of covariates followed three main principles: (1) referencing pertinent covariates identified in prior studies that used the NHANES database for gallstone research (15–17), (2) incorporating recognized risk factors documented in extensive reviews of gallstone disease (18), and (3) confirming that the chosen covariates were available within the NHANES dataset for analysis. This approach enabled the study to include covariates that were both evidence-based and readily accessible in the dataset.

### 2.5 Statistical analysis

All data analyses adhered to NHANES analytic guidelines and regulatory standards. NHANES-provided sample weights were included in our analysis of observational data. We applied NHANES-recommended sample weights using R package “survey” to ensure national representativeness. All analyses accounted for NHANES’ complex survey design by incorporating MEC examination weights that adjust for sampling variations and non-response. We examined differences in characteristics between participants with and without gallstones, using t-tests for continuous variables and chi-square tests for categorical variables. Logistic regression models estimated the odds ratios (ORs) and 95% confidence intervals (CIs) for the relationship between osteoporosis and gallstones. In Model 1, no adjustments for potential confounders were made. Model 2 was further adjusted for age, gender, race, and education level, while Model 3 additionally accounted for serum calcium levels and BMI. Model 4 included further adjustments for physical activity, drinking history, smoking history, hypertension, and diabetes. To assess the association between T-score and gallstone prevalence, we applied smoothing curve fitting (penalty spline method) and generalized additive model (GAM) regression. GAM was chosen because it can capture non-linear relationships, which is important in our study since we believe the relationship between T-scores and gallstone risk may not be linear. This approach allows us to model the data flexibly, without assuming a specific form for the relationship, providing a more accurate picture of how bone density relates to gallstone risk.

We employed mediation analysis to evaluate whether serum calcium and phosphorus influenced the association between osteoporosis and gallstones. This analysis partitioned the total association into direct and indirect effects, estimating the proportion mediated (19). Adjustments were made for sex, age, race, education, BMI, drinking history, and smoking status. Bootstrapping (1,000 iterations) was conducted to calculate 95% CIs. Subgroup analyses explored variation in the osteoporosis-gallstone association across age, gender, race, education, alcohol consumption, smoking status, hypertension, and diabetes, with the Wald test used to assess effect modifiers through multiplicative interactions. All statistical analyses were performed using R version 4.1.3, with a significance threshold of *p* < 0.05.

## 3 Results

### 3.1 Baseline characteristics

Following the application of inclusion and exclusion criteria, 3,375 participants with complete data on osteoporosis (Table 1), 2,765 participants with complete data on femoral neck BMD (Table 2) and 1,626 participants with complete data on lumbar spine BMD were included in the analysis (Supplementary Table S1).

**Table 1:** Demographic and clinic characteristics based on questionnaire of osteoporosis.

Characteristics	Total Adults (*N* = 3,375)	No gallstones (*N* = 2,907)	Gallstones (N = 468)	*p* value
Age (mean)	64.57 (9.10)	64.35 (9.05)	65.95 (9.35)	<0.001
Gender (%)				<0.001
Male	1776 (52.6)	1,619 (55.7)	157 (33.5)	
Female	1,599 (47.4)	1,288 (44.3)	311 (66.5)	
Race (%)				0.001
Non–Hispanic Black	864 (25.6)	773 (26.6)	91 (19.4)	
Mexican American	323 (9.6)	262 (9.0)	61 (13.0)	
Non–Hispanic White	1,431 (42.4)	1,218 (41.9)	213 (45.5)	
Other race	757 (22.4)	654 (22.5)	103 (22.0)	
Education (%)				0.441
Grade 0–12	615 (18.2)	532 (18.3)	83 (17.7)	
High school graduate	878 (26.0)	745 (25.6)	133 (28.4)	
College graduate above	1882 (55.8)	1,630 (56.1)	252 (53.8)	
BMI (mean)	30.21 (7.06)	29.81 (6.85)	32.66 (7.85)	<0.001
Total calcium (mg/dL)	9.30 (0.39)	9.30 (0.39)	9.27 (0.39)	0.096
Hypertension (%)				<0.001
No	1,518 (45.0)	1,353 (46.5)	165 (35.3)	
Yes	1857 (55.0)	1,554 (53.5)	303 (64.7)	
Diabetes (%)				<0.001
No	2,544 (75.4)	2,231 (76.7)	313 (66.9)	
Yes	831 (24.6)	676 (23.3)	155 (33.1)	
Drinking history (%)				0.065
No	2,756 (81.7)	2,359 (81.1)	397 (84.8)	
Yes	619 (18.3)	548 (18.9)	71 (15.2)	
Smoking history (%)				0.859
No	1,671 (49.5)	1,437 (49.4)	234 (50.0)	
Yes	1704 (50.5)	1,470 (50.6)	234 (50.0)	
Vigorous activity (%)				0.021
No	2,878 (85.3)	2,462 (84.7)	416 (88.9)	
Yes	497 (14.7)	445 (15.3)	52 (11.1)	
Osteoporosis (%)				<0.001
No	2,985 (88.4)	2,594 (89.2)	391 (83.5)	
Yes	390 (11.6)	313 (10.8)	77 (16.5)	

Means and percentages were adjusted for survey weights of NHANES. BMI, body mass index.

**Table 2:** Demographic and clinic characteristics based on femoral neck BMD.

Characteristics	Total adults (*N* = 2,765)	No gallstones (*N* = 2,408)	Gallstones (*N* = 357)	*p* value
Age(mean)	64.24 (9.00)	63.95 (8.93)	66.18 (9.22)	<0.001
Gender (%)				<0.001
Male	1,532 (55.4)	1,393 (57.8)	139 (38.9)	
Female	1,233 (44.6)	1,015 (42.2)	218 (61.1)	
Race (%)				0.011
Non–Hispanic Black	683 (24.7)	617 (25.6)	66 (18.5)	
Mexican American	266 (9.6)	221 (9.2)	45 (12.6)	
Non–Hispanic White	1,160 (42.0)	999 (41.5)	161 (45.1)	
Other race	656 (23.7)	571 (23.7)	85 (23.8)	
Education (%)				0.805
Grade 0–12	483 (17.5)	421 (17.5)	62 (17.4)	
High school graduate	705 (25.5)	609 (25.3)	96 (26.9)	
College graduate above	1,577 (57.0)	1,378 (57.2)	199 (55.7)	
BMI (mean)	29.33 (6.12)	29.03 (5.96)	31.34 (6.76)	<0.001
Total calcium (mg/dL)	9.30 (0.39)	9.30 (0.39)	9.29 (0.39)	0.47
Hypertension (%)				<0.001
No	1,299 (47.0)	1,167 (48.5)	132 (37.0)	
Yes	1,466 (53.0)	1,241 (51.5)	225 (63.0)	
Diabetes (%)				<0.001
No	2,130 (77.0)	1887 (78.4)	243 (68.1)	
Yes	635 (23.0)	521 (21.6)	114 (31.9)	
Drinking history (%)				0.184
No	2,249 (81.3)	1949 (80.9)	300 (84.0)	
Yes	516 (18.7)	459 (19.1)	57 (16.0)	
Smoking history (%)				0.647
No	1,375 (49.7)	1,202 (49.9)	173 (48.5)	
Yes	1,390 (50.3)	1,206 (50.1)	184 (51.5)	
Vigorous activity (%)				0.165
No	2,328 (84.2)	2018 (83.8)	310 (86.8)	
Yes	437 (15.8)	390 (16.2)	47 (13.2)	
T-score (mean)	−0.67 (1.36)	−0.65 (1.36)	−0.85 (1.38)	0.008
Degree of bone loss (%)				0.075
Normal	1,581 (57.2)	1,388 (57.6)	193 (54.1)	
Osteopenia	983 (35.6)	855 (35.5)	128 (35.9)	
Osteoporosis	201 (7.3)	165 (6.9)	36 (10.1)	

Means and percentages were adjusted for survey weights of NHANES. BMI, body mass index; BMD, bone mineral density.

In Table 1, 2,907 participants did not have gallstones, while 468 participants had gallstones. Statistically significant differences (*p* < 0.05) were observed between the two groups in terms of age, gender, race, BMI, physical activity, alcohol intake, diabetes, and hypertension. In Table 2, 2,408 participants without gallstones were compared to 357 participants with gallstones. Significant differences (*p* < 0.05) were found between the two groups with respect to age, gender, race, BMI, alcohol consumption, diabetes, hypertension, T-score, and degree of bone loss. In Supplementary Table S1, 1,418 participants without gallstones were compared to 208 participants with gallstones. Significant differences (*p* < 0.05) were found between the two groups with respect to age, gender, race, BMI, physical activity, and hypertension.

Compared to individuals without gallstones, those with gallstones were older. Among the gallstone group, a higher proportion were female compared to males. In terms of racial distribution, non-Hispanic white participants had a higher prevalence of gallstones compared to those without gallstones. Additionally, participants with gallstones had a higher proportion of overweight individuals compared to those without gallstones. A higher percentage of participants diagnosed with diabetes or hypertension were also found in the gallstone group compared to those without.

**Figure 2:**
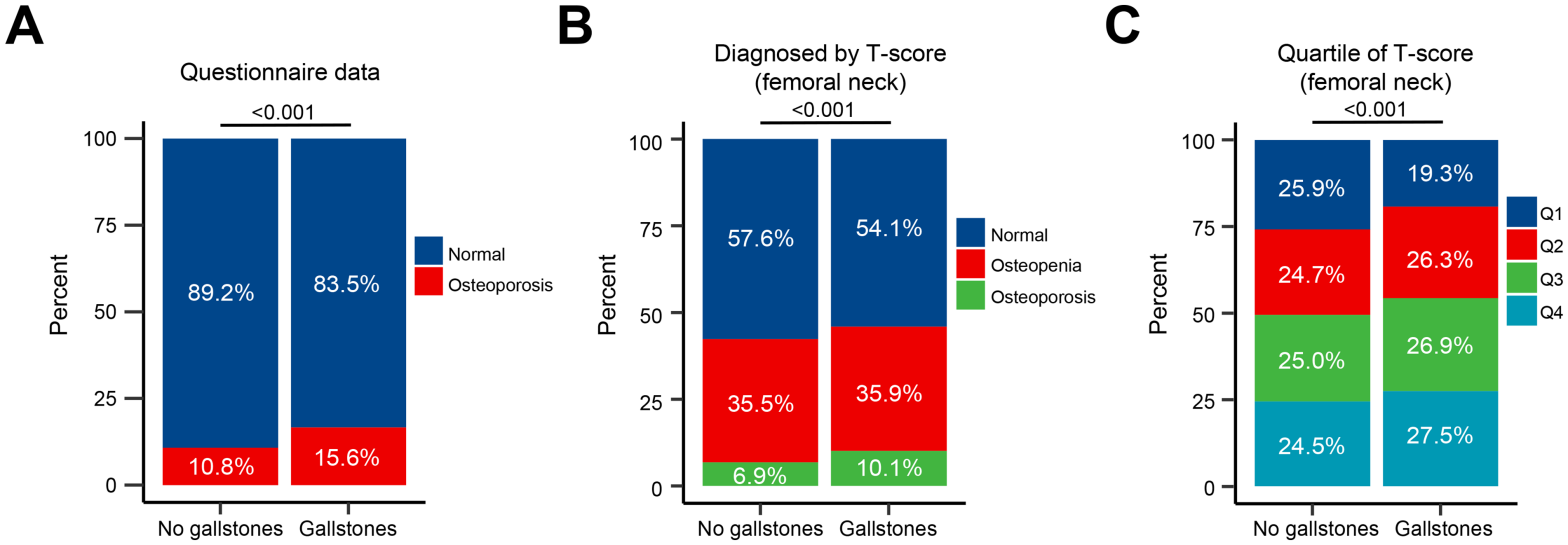
Association of osteoporosis with gallstone prevalence. **(A)** Proportion of osteoporosis in participants with and without gallstones, as indicated by questionnaire data. **(B)** Proportion of osteopenia and osteoporosis based on femoral neck T-scores in participants with and without gallstones. **(C)** Proportion of participants with gallstones across quartiles of femoral neck T-scores (Q1-Q4).

Furthermore, a significant increase in osteoporosis was observed among participants with gallstones, as indicated by the questionnaire data (*p* < 0.001) (Figure 2A). Individuals with gallstones also showed a higher incidence of osteopenia and osteoporosis, based on femoral neck T-scores (*p* < 0.001) (Figure 2B). Compared to those without gallstones, a larger proportion of individuals in the Q3/Q4 femoral neck T-score groups were found to have gallstones (*p* < 0.001) (Figure 2C). No significant differences were observed between the gallstone and non-gallstone groups in terms of lumbar spine T-scores or quartile classifications (Supplementary Figure S1).

### 3.2 Multivariate logistic regression analyses based on questionnaire data

We further investigated the association between osteoporosis and gallstones using a weighted logistic regression model. As shown in Table 3, the group without osteoporosis served as the reference. In Model 1, which was unadjusted for any covariates, individuals with osteoporosis exhibited a significantly higher risk of gallstones compared to the reference group (OR: 1.63, 95% CI: 1.24–2.13, *p* < 0.001). Model 2 adjusted for age, gender, race, and education level, and the association between osteoporosis and gallstone risk remained significant (OR: 1.50, 95% CI: 1.13–1.96, *p* < 0.001). In Model 3, which further adjusted for serum calcium levels and BMI, individuals with osteoporosis continued to show a significant increased risk of gallstones (OR: 1.69, 95% CI: 1.27–2.21, *p* < 0.001). Finally, Model 4 included additional adjustments for physical activity, alcohol intake, smoking history, hypertension, and diabetes. Even with these adjustments, osteoporosis remained significantly associated with an elevated risk of gallstones (OR: 1.52, 95% CI: 1.15–1.99, *p* < 0.001). Subgroup analyses revealed that the association between osteoporosis and increased gallstone risk was particularly pronounced in participants aged 65 years or older, as well as in males.

**Table 3:** Logistic regression of gallstone risk base on questionnaire data of osteoporosis.

	OR	95% CI		*p* value
		Lower limit	Upper limit	
Total population				
Model 1	1.63	1.24	2.13	<0.001
Model 2	1.50	1.13	1.96	<0.001
Model 3	1.69	1.27	2.21	<0.001
Model 4	1.52	1.15	1.99	<0.001
Male				
Model 1	2.65	1.32	4.94	<0.001
Model 2	2.36	1.16	4.47	0.01
Model 3	2.78	1.38	5.22	<0.001
Model 4	2.46	1.21	4.62	0.01
Female				
Model 1	1.02	0.75	1.38	0.87
Model 2	0.96	0.69	1.31	0.78
Model 3	1.08	0.79	1.47	0.62
Model 4	0.97	0.71	1.31	0.83
>65 years				
Model 1	1.68	1.06	2.57	0.02
Model 2	1.75	1.10	2.70	0.01
Model 3	1.60	1.00	2.47	0.04
Model 4	1.59	1.00	2.45	0.04
≤65 years				
Model 1	1.44	1.01	2.03	0.04
Model 2	1.37	0.95	1.94	0.08
Model 3	1.57	1.09	2.22	0.01
Model 4	1.38	0.96	1.96	0.07

OR, odds ratio; CI, confidence interval. Model 1: Unadjusted model. Model 2: Adjusted for age, gender, race/ethnicity, and education level. Model 3: Further adjusted for serum calcium levels and BMI based on Model 2. Model 4: Further adjusted for physical activity, drinking history, smoking history, hypertension, and diabetes based on Model 3.

### 3.3 Multivariate logistic regression analyses based on BMD data

**Figure 3:**
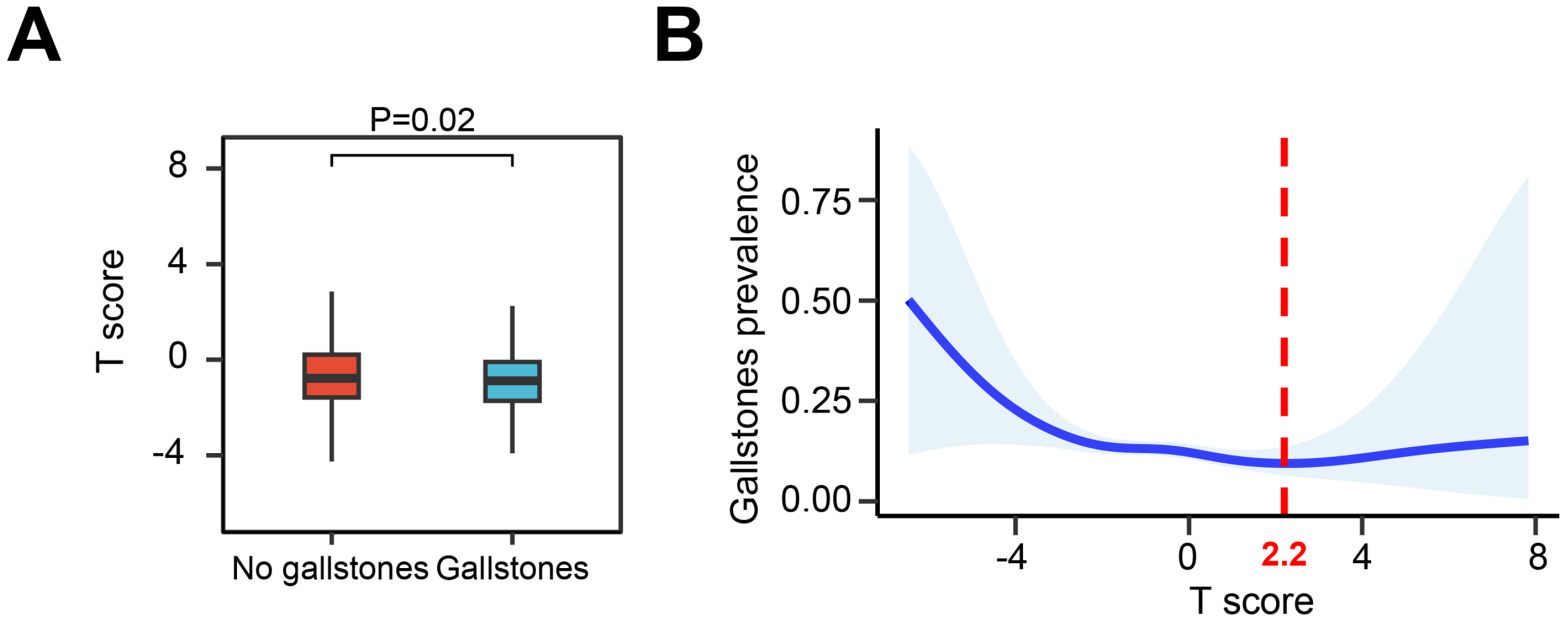
Relationship between femoral neck T-scores and gallstone risk. **(A)** Comparison of femoral neck T-scores in participants with and without gallstones. **(B)** Graphical representation of the non-linear relationship between femoral neck T-scores and gallstone risk using GAM and smooth curve fitting.

Subsequent analysis revealed that participants with gallstones had higher femoral neck T-scores compared to those without gallstones (Figure 3A). A generalized additive model (GAM) and smooth curve fitting were employed to further investigate the relationship between femoral neck T-scores and gallstone incidence. Our results demonstrated a non-linear association between *T*-score and gallstone risk (Figure 3B). Considering the effect of the saturation threshold between them, the likelihood natural ratio test found the best *T*-score threshold at 2.2.

Using DXA classification criteria, femoral neck T-scores greater than or equal to −1 were designated as the reference group. As shown in Table 4, the logistic regression results indicated that, compared to the reference group, participants with osteoporosis (T-score ≤ −2.5) had a 69% higher risk of gallstones in Model 1 (OR = 1.69, 95% CI: 1.05–2.30, *p* = 0.02), a 45% higher risk in Model 2 (OR = 1.45, 95% CI: 1.01–2.15, *p* = 0.05), a 123% higher risk in Model 3 (OR = 2.23, 95% CI: 1.47–3.32, *p* < 0.001), and a 67% higher risk in Model 4 (OR = 1.67, 95% CI: 1.11–2.46, *p* = 0.01). Next, participants were grouped into quartiles based on their femoral neck T-scores (Q1–Q4), with the highest quartile (Q1) as the reference group. Table 5 illustrates the relationship between T-score quartiles (Q1–Q4) and the risk of gallstones. In Models 1, 3, and 4, individuals in quartiles Q2–Q4 exhibited a significantly higher risk of gallstones compared to the reference group (OR > 1, *p* < 0.05). However, in Model 2, the association between T-score and gallstones was not statistically significant.

**Table 4:** Logistic regression of gallstone risk base on femoral neck BMD data.

T-score classification	OR	95% CI		*p* value
		Lower limit	Upper limit	
Model 1				
Normal	Ref			
Osteopenia	1.08	0.85	1.37	0.54
Osteoporosis	1.69	1.05	2.30	0.02
Model 2				
Normal	Ref			
Osteopenia	1.03	0.81	1.31	0.81
Osteoporosis	1.45	1.01	2.15	0.05
Model 3				
Normal	Ref			
Osteopenia	1.34	1.03	1.70	0.03
Osteoporosis	2.23	1.47	3.32	<0.001
Model 4				
Normal	Ref			
Osteopenia	1.13	0.89	1.44	0.32
Osteoporosis	1.67	1.11	2.46	0.01

*T-score classification as dependent variable.*

**Table 5:** Logistic regression of gallstone risk base on femoral neck BMD data.

Quartile of T-score	OR	95% CI		*p* value
		Lower limit	Upper limit	
Model 1				
Q1 (0.156, 5.248)	Ref			
Q2 (−0.798, 0.156)	1.43	1.03	1.99	0.03
Q3 (−1.596, −0.798)	1.44	1.04	2.01	0.02
Q4 (−4.633, −1.596)	1.50	1.08	2.09	0.02
Model 2				
Q1 (0.156, 5.248)	Ref			
Q2 (−0.798, 0.156)	1.35	0.97	1.90	0.07
Q3 (−1.596, −0.798)	1.34	0.96	1.88	0.08
Q4 (−4.633, −1.596)	1.37	0.99	1.94	0.06
Model 3				
Q1 (0.156, 5.248)	Ref			
Q2 (−0.798, 0.156)	1.77	1.26	2.49	0.001
Q3 (−1.596, −0.798)	1.96	1.39	2.77	<0.001
Q4 (−4.633, −1.596)	2.94	1.62	3.68	<0.001
Model 4				
Q1 (0.156, 5.248)	Ref			
Q2 (−0.798, 0.156)	1.50	1.07	2.10	0.02
Q3 (−1.596, −0.798)	1.55	1.12	2.17	0.01
Q4 (−4.633, −1.596)	1.64	1.18	2.30	0.003

BMD, bone mineral density; OR, odds ratio; CI, confidence interval. Model 1: Unadjusted model. Model 2: Adjusted for age, gender, race/ethnicity, and education level. Model 3: Further adjusted for serum calcium levels and BMI based on Model 2. Model 4: Further adjusted for physical activity, drinking history, smoking history, hypertension, and diabetes based on Model 3. Quartile of *T*-score as dependent variable.

Interestingly, we found no significant correlation between the lumbar spine T-scores and gallstone according to the logistic regression (Supplementary Table S2).

### 3.4 Subgroup and mediation analyses

**Figure 4:**
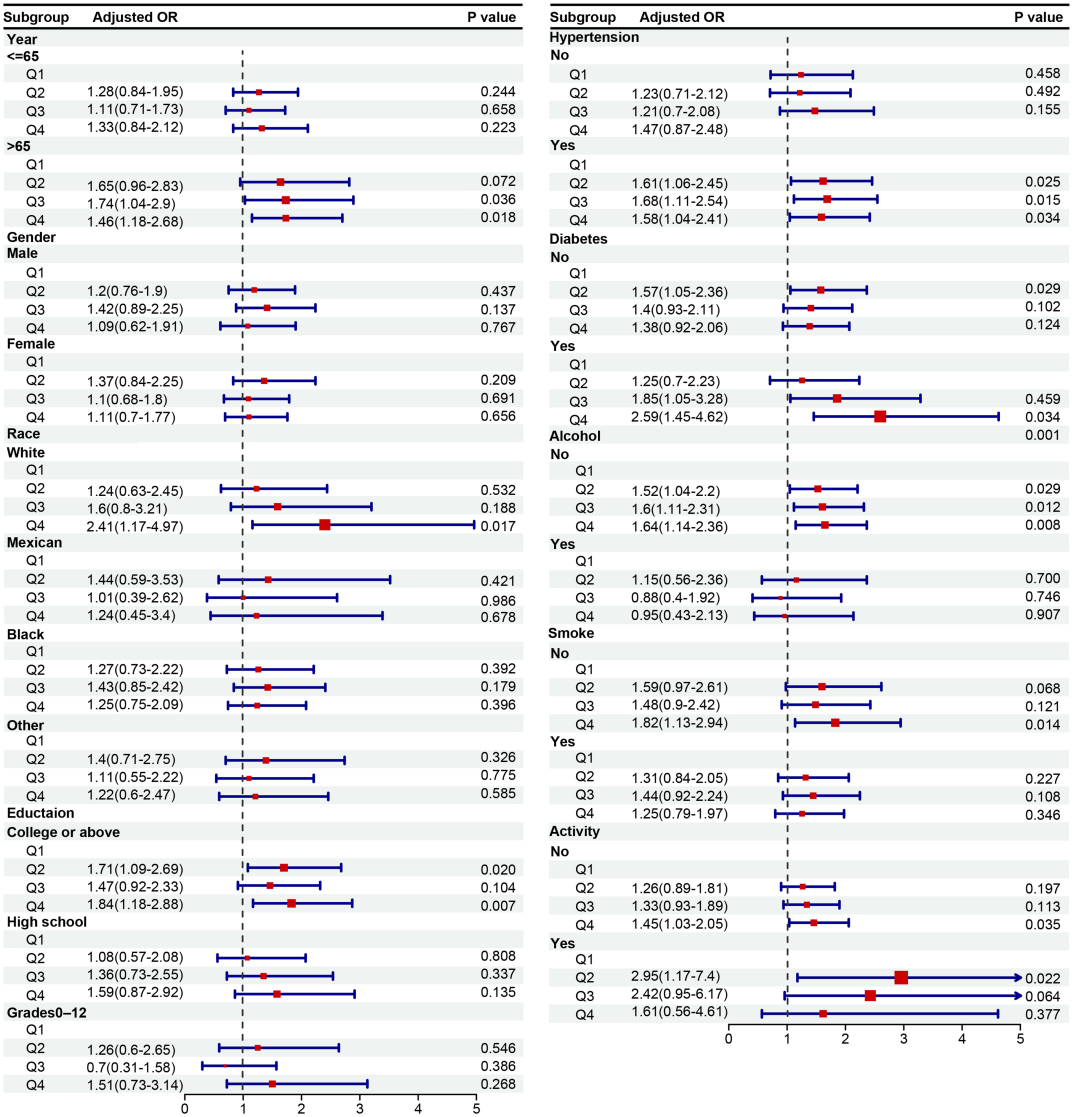
OR (95% CI) of gallstone disease according to quartile of T-score stratified by selected factors. OR, odds ratio; CI, confidence interval.

Subgroup analyses were conducted to explore whether the relationship between osteoporosis and gallstones was modified by factors such as age, gender, race, education level, smoking history, alcohol consumption, hypertension, and diabetes (Figure 4). After adjusting for confounding variables, a significant association was observed between the highest quartile of femoral neck T-scores (Q4) and an increased risk of gallstones, particularly among participants over 65 years old, non-Hispanic White individuals, those with a college education above, individuals with hypertension or diabetes, non-drinkers, non-smokers, and those with no vigorous physical activity.

Mediation analysis indicated that 5.4% of the association between osteoporosis and gallstones was mediated by serum calcium, and 3.3% was mediated by serum phosphorus (Supplementary Table S3).

## 4 Discussion

This broad, multiethnic, cross-sectional study targeting older adults in the United States aimed to explore the association between osteoporosis and gallstones. Based on the NHANES data from 2017 to 2020, we found a heightened risk of gallstones among individuals with osteoporosis, particularly among those over 65 years old, non-Hispanic White, individuals with hypertension, diabetes, non-drinkers, non-smokers, those who did not engage in vigorous physical activity, and those with a college education or higher. Notably, the T-score calculated from femoral neck BMD was a more effective predictor of osteoporosis-related gallstone risk than lumbar spine BMD. The generalized additive model and smooth curve fitting further revealed a nonlinear relationship between femoral neck T-scores and gallstone incidence, and serum calcium and phosphorus were identified as mediators of this association. These findings highlight the importance of assessing the severity of osteoporosis in older adults to predict their risk of developing gallstones.

To our knowledge, this is the first study to specifically assess the causal relationship between osteoporosis and gallstones using a U.S. cohort. A similar cohort study conducted in Taiwan found a 35% increased risk of gallstones among osteoporosis patients after 5 years of follow-up (9). While this finding aligns with our results, it is limited by its focus on an Asian population and the exclusion of important variables such as BMI, serum calcium, and lifestyle factors like smoking and alcohol consumption, which may also influence gallstone risk. Additionally, the diagnosis of osteoporosis in their study is based solely on public databases without specific bone density data. Furthermore, variations in sample size and ethnicity play a significant role, as the Taiwanese study focused on an Asian population, which may have different genetic and environmental influences on osteoporosis and gallstone formation compared to the U.S. cohort in our study. Additionally, differences in healthcare systems, access to medical care, and lifestyle factors such as diet and physical activity levels between Taiwan and the U.S. could contribute to observed differences in the relationship between osteoporosis and gallstones.

In our analysis, we included individual bone density measurements and their T-scores to further evaluate the relationship between osteoporosis and gallstones. We found that osteoporosis (severe bone loss, T-score ≤ −2.5) is a significant risk factor for gallstones, while osteopenia (moderate bone loss, T-score between −2.5 and − 1) did not show a significant association with gallstone risk. This underscores the importance of classifying osteoporosis severity when assessing gallstone risk. We also observed that femoral neck BMD, measured by DXA, was more strongly associated with gallstones than lumbar spine BMD. This discrepancy may be explained by bone hyperplasia in the lumbar spine, such as osteophytes or degenerative changes, which can distort bone density measurements (20). These changes add extra bone mass, potentially leading to an overestimation of bone density in older adults, which may account for the lack of a clear relationship between lumbar spine BMD and gallstones in our study.

Further subgroup analyses revealed significant variations in the osteoporosis-gallstone association based on demographic factors. Age is a key risk factor for both osteoporosis and gallstones, with osteoporosis being particularly prevalent in postmenopausal women. For instance, approximately 46% of women will experience at least one osteoporotic fracture after the age of 50 (21). The Sirmione study found that gallstone incidence in individuals aged 40–69 was four times higher than in younger individuals (1993). Moreover, a U.S. nationwide survey reported higher gallstone rates among non-Hispanic White individuals, who also have a higher risk of osteoporosis (22). These findings help explain the observed differences in the osteoporosis-gallstone relationship by age and race. Several studies suggest that alcohol consumption may reduce gallstone risk by lowering bile cholesterol saturation and increasing HDL cholesterol levels (23). Conversely, hypertension and diabetes, common in older adults, have been associated with an increased risk of both osteoporosis and gallstones (24–27). This may explain why we observed a stronger association between osteoporosis and gallstones in individuals with hypertension or diabetes, as well as in non-drinkers.

The direct association between osteoporosis and gallstones observed in our study can be explained by osteopontin (OPN), a pro-inflammatory cytokine involved in bone remodeling and resorption (28). High levels of OPN are known to increase bone resorption and, consequently, calcium and phosphate release into the bloodstream (29). OPN also plays a role in cholesterol gallstone formation, as it inhibits the nucleation of cholesterol crystals by binding to hydroxyapatite and calcium ions (30). Previous studies have shown that elevated OPN levels in the gallbladder wall are associated with gallstone formation (31). OPN deficiency can protect against cholesterol gallstone formation by altering biliary homeostasis and reducing hepatic cholesterol secretion (32). Our mediation analysis supports these findings, showing that blood calcium and phosphorus mediate part of the association between osteoporosis and gallstones. Alternative explanations for the osteoporosis-gallstone link include hormonal regulation and metabolic factors. Estrogen plays a dual role in both bone health and gallstone formation, as estrogen deficiency accelerates bone resorption while high estrogen levels contribute to bile cholesterol supersaturation (33). Obesity and insulin resistance also represent potential links, as both conditions are associated with osteoporosis and gallstone formation through inflammatory and lipid metabolism pathways (34).

This study has several strengths. It provides important clinical and public health insights into the prevention and management of gallstones in the U.S. population. The analyses were based on a large, nationally representative sample, making the findings broadly generalizable. Furthermore, we controlled for a range of potential confounders, including sociodemographic characteristics, BMI, and lifestyle factors such as smoking and alcohol use.

However, there are some limitations to consider. First, the cross-sectional nature of the NHANES dataset precludes establishing a causal relationship between osteoporosis and gallstones. Although we performed subgroup and mediation analyses to support the associations, longitudinal studies are necessary to confirm these findings. Second, despite adjusting for many potential confounders, residual confounding by unmeasured factors such as serum osteopontin, sex hormones, and other inflammatory markers cannot be ruled out. Additionally, dietary factors (e.g., calcium intake) and medication use (e.g., bisphosphonates, hormone therapy) are known to influence both osteoporosis and gallstone risk, but they were not included as covariates in our analysis due to limitations in data availability in the NHANES dataset. Future research should use prospective cohort designs or Mendelian randomization to establish causality more robustly. Lastly, this research was conducted in a U.S. population, and further validation in other populations is required to determine the generalizability of our findings.

## 5 Conclusion

In conclusion, this study using NHANES data finds a strong link between osteoporosis and a higher risk of gallstones, especially in older adults, non-Hispanic whites, and those with certain health conditions and lifestyle factors. Additionally, femoral neck bone density appears to be a stronger predictor of gallstone risk than lumbar spine bone density. Routine gallstone screening in individuals with osteoporosis, particularly those with additional risk factors may be beneficial.

## Data Availability

The datasets presented in this study can be found in online repositories. The names of the repository/repositories and accession number(s) can be found in the article/Supplementary material.
