# Supplementary material for: Association between osteoporosis and gallstone based on the National Health and Nutrition Examination Survey (NHANES) 2017–2020: a cross-sectional study
Source: Front Public Health. 2025 Apr 8;13:1562984. doi: 10.3389/fpubh.2025.1562984 (PMC12011783; doi:10.3389/fpubh.2025.1562984)
Supplement: Supplementary file 1 [file Table_1.docx]

Supplementary Material

# Supplementary Data

Supplementary Material should be uploaded separately on submission. Please include any supplementary data, figures and/or tables.

Supplementary material is not typeset so please ensure that all information is clearly presented, the appropriate caption is included in the file and not in the manuscript, and that the style conforms to the rest of the article.

# Supplementary Figures and Tables

## Supplementary Figures


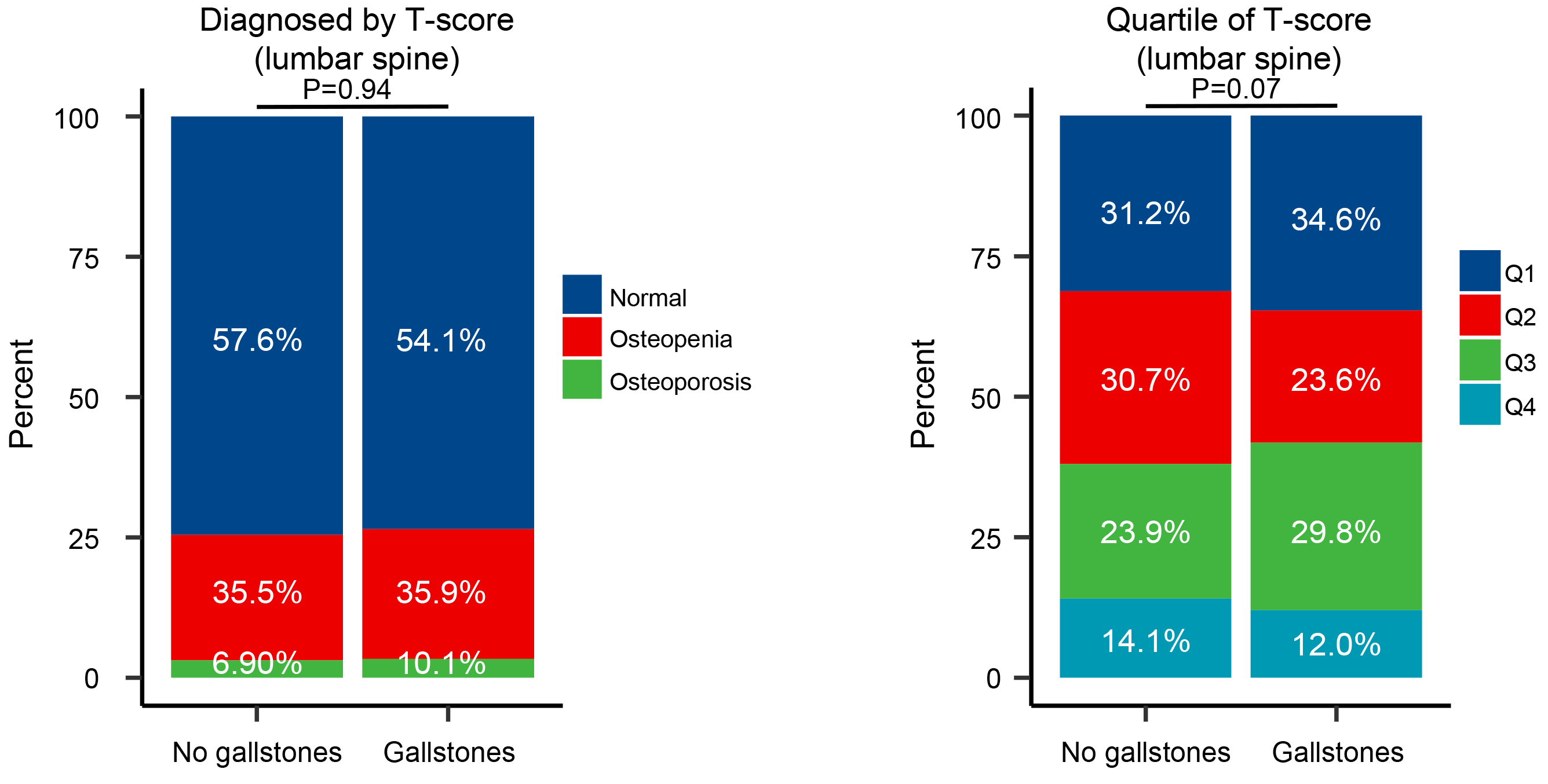


**Supplementary Figure 1.** Association of osteoporosis with gallstone prevalence. (A) Proportion of osteopenia and osteoporosis based on lumbar spine T-scores in participants with and without gallstones. (B) Proportion of participants with gallstones across quartiles of lumbar spine T-scores (Q1-Q4).

## Supplementary Figures

| Table S1. Demographic and clinic characteristics based on lumbar spine BMD. | | | | |
| --- | --- | --- | --- | --- |
| Characteristics | Total Adults (N = 1626) | No gallstones  (N = 1418) | Gallstones  (N = 208) | P value |
| Age(mean) | 62.83 (8.63) | 62.65 (8.57) | 64.10 (8.91) | 0.024 |
| Gender (%) |  |  |  | <0.001 |
| Male | 789 (48.5) | 738 (52.0) | 51 (24.5) |  |
| Female | 837 (51.5) | 680 (48.0) | 157 (75.5) |  |
| Race (%) |  |  |  | 0.076 |
| Non–Hispanic Black | 421 (25.9) | 378 (26.7) | 43 (20.7) |  |
| Mexican American | 178 (10.9) | 146 (10.3) | 32 (15.4) |  |
| Non–Hispanic White | 618 (38.0) | 539 (38.0) | 79 (38.0) |  |
| Other race | 409 (25.2) | 355 (25.0) | 54 (26.0) |  |
| Education (%) |  |  |  | 0.925 |
| Grade 0-12 | 276 (17.0) | 240 (16.9) | 36 (17.3) |  |
| High school graduate | 927 (57.0) | 811 (57.2) | 116 (55.8) |  |
| College graduate above | 423 (26.0) | 367 (25.9) | 56 (26.9) |  |
| BMI (mean) | 29.66 (6.62) | 29.32 (6.48) | 32.00 (7.13) | <0.001 |
| Total calcium (mg/dL) | 9.30 (0.38) | 9.30 (0.38) | 9.28 (0.39) | 0.543 |
| Hypertension (%) |  |  |  | 0.001 |
| No | 781 (48.0) | 703 (49.6) | 78 (37.5) |  |
| Yes | 845 (52.0) | 715 (50.4) | 130 (62.5) |  |
| Diabetes (%) |  |  |  | 0.054 |
| No | 1254 (77.1) | 1105 (77.9) | 149 (71.6) |  |
| Yes | 372 (22.9) | 313 (22.1) | 59 (28.4) |  |
| Drinking history (%) |  |  |  | 0.351 |
| No | 1359 (83.6) | 1180 (83.2) | 179 (86.1) |  |
| Yes | 267 (16.4) | 238 (16.8) | 29 (13.9) |  |
| Smoking history (%) |  |  |  | 0.936 |
| No | 868 (53.4) | 758 (53.5) | 110 (52.9) |  |
| Yes | 758 (46.6) | 660 (46.5) | 98 (47.1) |  |
| Vigorous activity (%) |  |  |  | <0.001 |
| No | 1418 (87.2) | 1418 (100.0) | 0 (0.0) |  |
| Yes | 208 (12.8) | 0 (0.0) | 208 (100.0) |  |
| T-score (mean) | 0.00 (1.50) | 0.00 (1.51) | 0.03 (1.50) | 0.836 |
| Degree of bone loss (%) |  |  |  | 0.949 |
| Normal | 1210 (74.4) | 1057 (74.5) | 153 (73.6) |  |
| Osteopenia | 365 (22.4) | 317 (22.4) | 48 (23.1) |  |
| Osteoporosis | 51 (3.1) | 44 (3.1) | 7 (3.4) |  |
| Means and percentages were adjusted for survey weights of NHANES. BMI: body mass index; BMD: bone mineral density. | | | | |

| Table S2. Logistic regression of gallstone risk base on lumbar spine BMD data. T-score classification as dependent variable. | | | | | |
| --- | --- | --- | --- | --- | --- |
|  | T-score classification | OR | 95%CI | | P value |
|  |  |  | Lower limit | Upper limit |  |
| Model1 |  |  |  |  |  |
|  | Normal | Ref |  |  |  |
|  | Osteopenia | 1.05 | 0.73 | 1.47 | 0.80 |
|  | Osteoporosis | 1.10 | 0.45 | 2.33 | 0.82 |
| Model2 |  |  |  |  |  |
|  | Normal | Ref |  |  |  |
|  | Osteopenia | 1.02 | 0.71 | 1.43 | 0.91 |
|  | Osteoporosis | 1.07 | 0.43 | 2.30 | 0.89 |
| Model3 |  |  |  |  |  |
|  | Normal | Ref |  |  |  |
|  | Osteopenia | 1.36 | 0.94 | 1.94 | 0.09 |
|  | Osteoporosis | 1.68 | 0.67 | 3.64 | 0.22 |
| Model4 |  |  |  |  |  |
|  | Normal | Ref |  |  |  |
|  | Osteopenia | 1.09 | 0.76 | 1.53 | 0.65 |
|  | Osteoporosis | 0.44 | 2.31 | 2.46 | 0.85 |
| BMD, bone mineral density; OR, odds ratio; CI, confidence interval.  Model 1 was Unadjusted model;  Model 2 was adjusted for age, gender, race/ethnicity, and education level;  Model 3 was adjusted for serum calcium levels and BMI;  Model 4 was adjusted for physical activity, drinking history, smoking history, hypertension, and diabetes. | | | | | |

| Table S3. Mediation analysis of the association between osteoporosis and gallstone by serum calcium and phosphorus. | | | | |
| --- | --- | --- | --- | --- |
|  | OR | 95% CI | | P value |
|  |  | Lower limit | Upper limit |  |
| Serum calcium |  |  |  |  |
| Direct effect | 1.073 | 1.029 | 1.116  2 | <0.001 |
| Indirect effect | 1.076 | 1.031 | 1.116 | <0.001 |
| Total effect | 1.070 | 1.028 | 1.116 | <0.001 |
| Serum phosphorus |  |  |  |  |
| Direct effect | 1.069 | 1.027 | 1.10  2 | <0.001 |
| Indirect effect | 1.085 | 1.031 | 1.182 | 0.05 |
| Total effect | 1.071 | 1.029 | 1.116 | <0.001 |
| OR: odds ratio; CI: confidence interval.  Results were adjusted for sex, age, race, and education. We performed 1000 iterations for bootstrapping to estimate 95% CIs. | | | | |
